# Application of neuromodulation techniques in irritable bowel syndrome

**DOI:** 10.3389/fnins.2026.1792685

**Published:** 2026-05-11

**Authors:** Yingchun Zhang, Liyuan Gao, Xiaoguang Zhang, Wenkai Sun, Meng Wang, Tingting Zhang, Xiaoyu Wang

**Affiliations:** 1 Department of Gastroenterology, Zhangjiagang Traditional Chinese Medicine Hospital Affiliated to Nanjing University of Chinese Medicine, Suzhou, China; 2 Department of Pharmacy, Zhangjiagang Traditional Chinese Medicine Hospital Affiliated to Nanjing University of Chinese Medicine, Suzhou, China; 3 Department of Neurology, First Affiliated Hospital of Dalian Medical University, Dalian, China

**Keywords:** brain-gut axis, electroacupuncture, irritable bowel syndrome, neuromodulation, sacral nerve stimulation (SNS)

## Abstract

Irritable bowel syndrome (IBS) is a gut-brain interaction disorder characterized by abdominal pain/abdominal discomfort accompanied by changes in bowel motility. Its pathogenesis involves the interaction of multiple factors, including abnormalities of the brain-gut axis, intestinal microbiota dysbiosis, and visceral hypersensitivity. Traditional treatment strategies mainly focus on symptomatic relief, which have limitations such as insufficient targeting and significant side effects. Neuromodulation techniques, as an emerging treatment modality, modulate the central or peripheral nervous system through electrical and electromagnetic means, targeting key pathways of the brain-gut axis. These techniques can regulate gut motility and suppress inflammatory responses, thereby alleviating IBS symptoms. Currently, several techniques are widely applied, including electroacupuncture (EA), Sacral Nerve Stimulation (SNS), transcutaneous auricular vagus nerve stimulation (taVNS), and transcutaneous electrical nerve stimulation (TENS). Multiple clinical trials have confirmed their effectiveness in relieving abdominal pain, improving bowel dysfunction, and enhancing quality of life. However, there are still issues such as significant individual differences and insufficient long-term efficacy data. Future research should focus on the development of personalized treatment plans, exploration of combination therapy strategies, and technological innovation to further enhance their clinical value.

## 1 Irritable bowel syndrome (IBS)

### 1.1 Definition and epidemiology of IBS

IBS is a common gut-brain interaction disorder, rather than a purely functional gastrointestinal disease. Its core features are recurrent abdominal pain or abdominal discomfort, accompanied by changes in bowel motility (such as altered frequency or stool form), with symptoms lasting for more than 6 months in the absence of evidence of organic pathology (such as inflammation, infection, or neoplasm) (Vasant et al., 2021).

The global average prevalence of IBS in the general population is reported to range from 4 to 10% (Greer and Sultan, 2025). The impact of diagnostic criteria on prevalence is particularly significant (based on population data from the United States, Canada, and the United Kingdom, the prevalence of IBS diagnosed by Rome III is approximately twice that of Rome IV) (Huang et al., 2023; Sperber et al., 2017; Black and Ford, 2020; Palsson et al., 2020).

### 1.2 Diagnosis and subtyping of IBS

The current diagnosis of IBS generally uses the Rome IV criteria. The relevant symptoms must have been present for at least 6 months, with the criteria met in the last 3 months. In addition, it is necessary to exclude organic diseases that can explain the symptoms (such as inflammatory bowel disease, colorectal cancer, celiac disease, etc.) through routine clinical examinations (such as physical examination, laboratory tests, endoscopy, etc.) (Enck et al., 2016).

Referring to the Bristol Stool Form Scale (Lewis and Heaton, 1997), IBS can be classified into four subtypes, which helps guide treatment: IBS with diarrhea (IBS-D), IBS with constipation (IBS-C), IBS with mixed bowel habits (IBS-M) and Unspecified IBS (IBS-U) (Vasant et al., 2021).

### 1.3 Pathogenesis of IBS

The pathogenesis of IBS is complex and involves the interaction of multiple factors. Current research mainly focuses on abnormalities of the brain-gut axis, intestinal microbiota dysbiosis, visceral hypersensitivity, intestinal barrier dysfunction, and gut motility disorders (Sperber et al., 2021).

#### 1.3.1 Brain-gut axis dysfunction (core mechanism)

The brain-gut axis mechanism of IBS is a complex, bidirectional interaction involving the intestinal environment, the central nervous system (CNS), and the neural, endocrine, and immune pathways connecting them (Drossman and Hasler, 2016). Within the gut, factors such as microbiota dysbiosis, abnormal mucosal immune function, and increased intestinal permeability can alter gut-derived signaling, generating visceral hypersensitivity (Simrén et al., 2018). These signals are transmitted to the brain via the vagal afferents and to the spinal cord via multiple autonomic afferents, activating brain regions associated with pain perception, mood control, and autonomic nervous function, such as the anterior cingulate cortex, insula, and amygdala. This leads to visceral hypersensitivity and emotional disturbances (Mayer et al., 2019). At the same time, dysfunction of the CNS, including central sensitization and autonomic nervous system dysregulation, further impacts gut motility and secretory functions. For example, Stress and anxiety can exacerbate IBS symptoms by activating the hypothalamic–pituitary–adrenal (HPA) axis and excessive sympathetic activation, which increases gut sensitivity and motility. In addition, impaired brain regulation of the gut results in abnormal transmission and processing of gut signals, creating a vicious cycle that further intensifies IBS symptoms (Mearin et al., 2016). Dysfunction of the brain-gut axis causes hypersensitivity and motor dysfunction in the gut of IBS patients, exacerbating symptoms.

#### 1.3.2 Intestinal dysbiosis

Intestinal dysbiosis contributes to IBS through multiple mechanisms. First, IBS patients often exhibit reduced gut microbiota diversity, with a decrease in beneficial bacteria and an increase in harmful bacteria. This imbalance affects the stability of the intestinal microbiome, thereby impacting gut function (Bhattarai et al., 2017). Second, the levels of short-chain fatty acids (SCFAs) are reduced, which weakens the intestinal barrier function, increases intestinal permeability, triggers inflammatory responses, and exacerbates IBS symptoms. Additionally, alterations in tryptophan metabolism affect neurotransmitter levels, leading to hypersensitivity of gut sensation and motor dysfunction. Intestinal dysbiosis also affects the central nervous system via the vagus nerve, modulating emotions and behaviors, and is associated with the emotional disturbances and abdominal pain in IBS patients (Bonaz et al., 2017). In terms of immune activation, the number of inflammatory cells in the intestinal mucosa of IBS patients increases, along with elevated levels of inflammatory mediators, further worsening intestinal inflammation and symptoms. Dietary and lifestyle factors, such as the intake of high-FODMAP foods and chronic psychological stress, can also lead to microbial imbalance and affect gut function (Zhao et al., 2024).

#### 1.3.3 Visceral hypersensitivity

Visceral hypersensitivity exacerbates IBS symptoms directly by lowering the pain threshold in the gastrointestinal tract at the level of peripheral afferent nerve endings (Simrén et al., 2018). In five different cohorts, the gastrointestinal symptoms of IBS patients were found to progressively worsen with the increase in visceral hypersensitivity. This trend was consistent across various testing sites (such as the rectum and colon) and IBS subtypes, with statistical significance. Importantly, this association remained significant after adjustment for central nervous system-related factors, indicating that the core manifestation of visceral hypersensitivity is independent of central sensitization-related processes.

#### 1.3.4 Intestinal barrier dysfunction

There is a close relationship between IBS and abnormalities in the structure of the intestinal barrier. All subtypes of IBS are characterized by structural abnormalities of the intestinal barrier, which are mainly manifested as abnormalities in tight junction (TJ) proteins and disordered regulation of the cytoskeleton, and are directly related to symptoms (González-Castro et al., 2017; Martínez et al., 2013). In terms of tight junction proteins, IBS patients (especially those with IBS-D) have structural and localization abnormalities of TJ proteins in the small intestinal epithelium: Occludin (OCLN) is translocated from the cell membrane to the cytoplasm after dephosphorylation (Martínez et al., 2013). In the zonula occludens (ZO) family, the expression of ZO-1 is reduced and redistributed from the cell apex to the cytoplasm (Martínez et al., 2012). In the Claudins family, IBS-D patients have upregulated Claudin-2 (CLDN-2) in the jejunum and reduced Claudin-1(CLDN-1) and Claudin-4 (CLDN-4) in the small intestinal mucosa (Kong et al., 2007). These changes lead to widened cell gaps, increasing the likelihood of luminal contents penetrating the mucosa (Bertiaux-Vandaële et al., 2011). Abnormal regulation of the cytoskeleton is equally important. IBS-D patients have increased expression of Myosin Light Chain Kinase (MLCK) and increased phosphorylation of Myosin Light Chain (MLC), which triggers contraction of the actin cytoskeleton and further disrupts the tight junction structure (Martínez et al., 2013). In addition, structural abnormalities of the jejunal epithelium (such as widened cell gaps) are significantly correlated with the severity of abdominal pain, bowel movement frequency, and stool consistency, suggesting that abnormalities in the structure of the intestinal barrier are an important basis for the development of IBS symptoms (Martínez et al., 2013).

#### 1.3.5 Gut motility disorders

Gut motility disorders are a core pathological basis of IBS, directly driving symptoms through abnormal whole-gut transit and disordered neuroendocrine regulation: IBS patients exhibit high-amplitude propulsive contractions (HAPC) in the gut during fasting, at a frequency 2–3 times that of healthy individuals, leading to a significant reduction in small bowel transit time (average 35 min for IBS-D vs. 68 min for healthy individuals), directly causing diarrhea. Postprandial motility shows subtype differentiation: in IBS-D, colonic mass movements are prematurely activated (30 min vs. 60 min), while in IBS-C, colonic motility amplitude is reduced by 50%, forming the core differences for diarrhea/constipation (Palsson et al., 2016).

Neuroendocrine regulation imbalance: IBS-D patients have a 40% increase in the density of enterochromaffin cells and a 2.3-fold increase in 5-HT release, which accelerates gut transit through the 5-HT3 receptor; IBS-C patients have downregulated 5-HT4 receptor expression, inhibiting colonic motility. Abnormalities of the brain-gut axis led to hyperactive IBS-D or inhibited IBS-C postprandial gastrocolic reflex, with small bowel transit time shortening to 28 min under stress (Sandler et al., 2002).

In summary, the pathogenesis of IBS is the result of the interaction of multiple factors, with the brain-gut axis as the core link. The gut microbiota, visceral sensitivity, barrier function, and motility disorders interact with each other, collectively leading to symptoms such as abdominal pain and abnormal bowel movements (Sperber et al., 2021).

### 1.4 Treatment approaches for IBS

The treatment of IBS typically follows a comprehensive approach that integrates dietary modifications, pharmacotherapy, and psychological interventions. In terms of diet, the low-FODMAP diet has been proven effective for the majority of IBS patients. It alleviates symptoms such as abdominal pain and bloating by reducing the production of fermentable substances in the gut (Black et al., 2022). In addition, eliminating specific foods like lactose and gluten can also help some patients improve their symptoms. Pharmacotherapy is usually selected based on the type of symptoms: for IBS-D, drugs such as loperamide are commonly used; whereas for IBS-C, laxatives like polyethylene glycol and stimulant laxatives are primarily used. For patients with more severe symptoms, neuroregulatory drugs such as antidepressants can also be used to relieve abdominal pain and improve mood. Meanwhile, psychological interventions such as cognitive-behavioral therapy and gut-directed hypnotherapy have also shown positive effects in the treatment of IBS, helping to improve patients’ quality of life (Vasant et al., 2021; Camilleri and Boeckxstaens, 2023).

However, the limitations of traditional treatment approaches for IBS are significant. There is a lack of targeting of core mechanisms such as gut-brain interaction and intestinal barrier abnormalities, with a focus mainly on symptomatic treatment targeting single symptoms like diarrhea or constipation. For example, although loperamide improves diarrhea and urgency in IBS-D, it is ineffective for abdominal pain and bloating, and fails to cover the entire symptom complex (Simrén and Tack, 2018). Side effects are pronounced, antispasmodics cause dry mouth and blurred vision in 25–30% of patients, and polyethylene glycol and other laxatives induce abdominal pain in 15–20% of patients, affecting adherence (Ford et al., 2014). The complex pathophysiological mechanisms are often overlooked. For instance, approximately 25% of IBS-D cases are actually bile acid diarrhea, yet they are missed due to the lack of 7αC4 testing (Bajor et al., 2015). Dietary modifications such as the low-FODMAP diet are effective in the short term but require professional guidance, and long-term use can lead to imbalances in gut microbiota, such as a reduction in bifidobacteria (Staudacher et al., 2017). Moreover, there is a general neglect of psychological comorbidities and interventions such as cognitive-behavioral therapy, which are severely underutilized (Black et al., 2020a). Therefore, while improving comprehensive individualized treatment plans, there is also a need to seek more precise, effective, and low-side-effect treatment options.

## 2 Neuromodulation

Neuromodulation refers to the use of implantable or non-implantable technologies, employing physical means (such as electrical, magnetic, optical, or ultrasound) or chemical methods to excite, inhibit, or modulate the transmission of signals in neurons or neural networks in the central, peripheral, and autonomic nervous systems, either locally or remotely. This biomedical engineering technology aims to improve patients’ quality of life and enhance their neurological functions (Xie and Zhang, 2022).

### 2.1 Development and subtyping of neuromodulation techniques in IBS

The history of using neuromodulation techniques for therapeutic purposes is long-standing. As early as 46 AD, the Roman physician Scribonus Largus used the black torpedo fish to treat headaches and gout, which is the earliest documented use of local electroanalgesia that can be traced back to Everaert et al. (2001). Melzack and Wall (1965) proposed the “gate control theory of pain,” laying the foundation for the birth of modern neuromodulation techniques. Research during this period mainly focused on pain management and basic studies of the enteric nervous system. Entering the 1990s, with a deeper understanding of gastrointestinal dysfunction, electrical stimulation techniques began to be applied to the treatment of gastrointestinal motility disorders and IBS. In particular, spinal cord stimulation (SCS) and sacral nerve stimulation (SNS) gradually emerged as effective methods for treating IBS (Chen, 2023). Vagus nerve stimulation (VNS) was initially developed as a treatment for drug-resistant epilepsy and depression. Its principle is based on modulating the function of the nervous system through electrical stimulation of the vagus nerve. As the role of the vagus nerve in regulating gastrointestinal function became better understood, VNS also began to be applied in the treatment of gastrointestinal diseases (Bonaz et al., 2017). In 2000, scientists proposed a non-invasive method to stimulate the auricular branch of the vagus nerve, known as transcutaneous auricular vagus nerve stimulation (taVNS). This method utilizes the distribution of the vagus nerve in the ear to perform non-invasive neuromodulation, thereby avoiding the risks associated with surgical implantation of VNS (Wang et al., 2022). Currently, taVNS is gradually being applied to the treatment of various diseases, including anxiety, depression, and pain management, and is beginning to show potential in the treatment of IBS (Shi et al., 2021). In the 2000s, electroacupuncture (EA) emerged as an innovative form of modern acupuncture treatment and gradually became a new method for treating IBS. EA modulates the nervous system by applying electrical current to specific acupuncture points, regulating gut motility and visceral hypersensitivity, and improving symptoms such as abdominal pain and constipation (Table 1) (Han et al., 2018).

**Table 1:** The equipment parameters and treatment plans of the neuromodulation technology applied in the treatment of irritable bowel syndrome.

Technique	Site	Device	Parameters	Protocol	Year
EA	Acupuncture at Guanyuan (CV 4), Zhongwan (CV 12), Tianshu (ST 25), Dachangshu (BL 25), Zusanli (ST 36), Shangjuxu (ST 37)	EA	1 Hz in frequency, 4–6 mA in current intensity 30 min	30 min, 6 times a week, for 4 weeks	Sun et al. (2021)
	Homotopic acupoints Tianshu (ST25) and Wailing (ST26), Heterotopic acupoints Zusanli (ST36) and Shangjuxu (ST37)	EA	2/100 Hz, noxious group has a pain threshold of 130%, non-noxious stimuli have a pain threshold of 70%	30 min,14 sessions of treatment, twice per week for 7 weeks	Peng et al. (2018)
	Bilateral Tianshu (ST25) and Shangjuxu (ST37)	EA Mox	EA: 2 Hz,3.0 mA Mox: acupoint surface temperature of 46 °C ± 1 °C.	30 min, 6 times a week, for 4 weeks	Zhao et al. (2015)
	Tianshu (ST25), Fubai (SP14), Zusanli (ST36), Shangjuxu (ST37), Shenshu (BL23), Dachangshu (BL25), Zhongliao (BL32), Xiagliao (BL33)	EA	2 Hz/15 Hz	30 min, 5 times a week, for 2 weeks	Zhou et al. (2014)
	Quchi (LI11), Shangjuxu (ST37), Tianshu (ST25), Dachangshu (BL25)	EA	15 Hz	16 treatments in 4 weeks (10 in first 2 weeks, 6 in last 2 weeks)	Zheng et al. (2016)
	Bilateral Neiguan (PC6), Shenmen (HT7), Taichong (LR3), Sanyinjiao (SP6), Zusanli (ST36), Shangjuxu (ST37), plus Baihui (GV20), Yintang (EX-HN3)	EA	2 Hz,0.5–1.5 mA	30 min, once weekly for 10 sessions	Mak et al. (2019)
SNM	Sacral Nerve	Test electrodes; external neurostimulator; permanent neurostimulator and leads.	14 Hz, 210 μs, 0.1–4.0 V	Open or close randomly based on the group, for each stage for 1 month	Fassov et al. (2014)
	Sacral Nerve	Permanent sacral neuromodulation device	Based on the individual response of the patient	Permanent implantation	Fassov et al. (2025)
	Sacral Nerve	Test electrodes, external neurostimulator, permanent neurostimulator and leads.	Sub-sensory: 90% of the optimal perception intensity. Supra-sensory: parameters set based on the patient’s optimal perception response.	Participants were randomly assigned to receive sub-sensory stimulation or to be deprived of it, for 2 weeks each stage	Fassov et al. (2019)
ta-VNS	Bilateral auricular cymba concha (innervated by the auricular branch of the vagus nerve)	Transcutaneous electrical stimulator (SNM-FDC01, Ningbo Meda Medical Equipment Co., Ltd.), with surface electrodes.	25 Hz, 0.5 ms, 2 s on/3 s off, 0–2 mA.	Bilateral cymba concha, 30 min twice daily at 08:00 and 20:00 for 4 weeks.	Shi et al. (2021)
	External ear(auricular branch of the vagus nerve)	NeuroStim System®	3.2 V rectangular pulses, 1 Hz/10 Hz alternating, 2 h on/2 h off.	5 days weekly for 4 weeks.	Krasaelap et al. (2020)
TENS	Hegu (LI4, right hand), Zusanli (ST36, left lower limb)	Transcutaneous electrical stimulation device (model not specified).	LI4: 100 Hz, 0.1 s/0.4 s train, 0.5 ms; ST36: 25 Hz, 2 s/3 s train, 0.5 ms.	TEA: Hegu (LI4, right hand) & Zusanli (ST36, left leg), 30 min twice daily after meals for 1 month.	Hu et al. (2022)
	Bilateral Shangjuxu (ST37) and Tianshu (ST25)	Neuromuscular electrical stimulator (HANS-100, Nanjing Jisheng Medical Technology Co., Ltd.)	2–100 Hz	Combined: DHC 80 mg orally twice daily for 4 weeks plus NMES (bilateral Shangjuxu (ST37) and Tianshu (ST25), 20 min twice weekly for 4 weeks).	Wang and Liu (2018)
	Abdominal and dorsal paravertebral area (T9-L2 level)	Transcutaneous interferential current therapy device (Ito EU-940, Germany)	Carrier 4 kHz, beat 80–150 Hz, 15–25 mA.	Abdomen and T9-L2 paraspinal, four crossed electrodes, 15 min three times weekly for 4 weeks.	Çoban et al. (2012)
	Bilateral Zusanli (ST36), Bilateral Neiguan (PC6)	A watch-sized electrical stimulator (Transtimulation Research Inc.)	TEA ST36 or PC6: 100 Hz, 0.5 ms, on 0.1 s/off 0.4 s; TEA ST36 or PC6: 25 Hz, 0.5 ms, on 2 s/off 3 s	Five 15-min randomized sessions on separate days: TEA/ST36-100 Hz, bilateral ST36; TEA/ST36-25 Hz, bilateral ST36; TEA/PC6-100 Hz, bilateral PC6; TEA/PC6-25 Hz, bilateral PC6.	Nojkov et al. (2024)
SCS	T5-T8 level of the dorsal epidural space	Quad-pole electrode (Quad-plus, Medtronic); pulse generator (Itrel-3, Medtronic).	50 Hz, 200–500 μs pulse width, 1.3–3.3 V amplitude.	Stimulation 8–12 h daily, total 28 w (2 w run-in, 6 w crossover, 12 w continuous, 2 w washout).	Lind et al. (2015)
TMS	Motor cortex (anal sphincter area), midline 2–3 cm in front of the vertex	Magstim 200 (monophasic single-pulse) and Magstim Super Rapid (biphasic repetitive)	10 Hz rTMS	600 pulses	Algladi et al. (2015)
LSMS	Lumbosacral vertebrae (midline ~5 cm above natal cleft; or 2–3 cm left/right of midline at iliac crests level)	Magstim 200 (monophasic single-pulse) and Magstim Super Rapid (biphasic repetitive)	1 Hz rLSMS	600 pulses	Algladi et al. (2015)

EA, Electroacupuncture; LSMS, Lumbosacral Magnetic Stimulation; Mox, Moxibustion; SCS, Spinal cord stimulation; SNM, Sacral Neuromodulation; ta-VNS, Transcutaneous Auricular Vagal Nerve Stimulation; TEA, Transcutaneous Electrical Acustimulation; TENS, Transcutaneous Electrical Nerve Stimulation; TMS, Transcranial Magnetic Stimulation.

### 2.2 Mechanisms of neuromodulation in treating IBS

Neuromodulation techniques improve visceral hypersensitivity, abnormal gut motility, and pain symptoms in IBS by targeting key pathways of the gut-brain axis (Aljeradat et al., 2024). Neuromodulation blocks the“gut-spinal cord-brain”pain amplification loop, reducing the gut hyperresponsiveness to mechanical and chemical stimuli. For example, EA inhibits the upload of nociceptive signals by reducing the phosphorylation of p38 and the expression of c-Fos in the spinal dorsal horn. It can also downregulate colonic 5-HT3 receptors, blocking 5-HT-mediated primary afferent sensitization (Chu et al., 2011; Xu et al., 2010). The core network mechanism of regulating bidirectional communication of the brain-gut axis takes the vagus nerve as a key hub, which realizes dynamic interaction between the central system and the gut through 80% afferent fibers and 20% efferent fibers. Afferent fibers convey information about the gut inflammatory status and microbiota signals to brain regions such as the limbic system and prefrontal cortex via the nucleus tractus solitarius (NTS), mediating pain and mood control. Efferent fibers, on the other hand, regulate gut motility, secretion, and barrier function through the enteric nervous system (Bonaz et al., 2021). Inhibiting low-grade intestinal inflammation as a core pathological basis, neuromodulation is achieved through multiple pathways: downregulating pro-inflammatory factors such as TNF-α and IL-6, upregulating anti-inflammatory factors such as IL-10, inhibiting macrophage activation, and suppressing mast cell release of histamine and tryptase (Shi et al., 2021). The cholinergic anti-inflammatory pathway is activated, with acetylcholine acting on the *α*7 nicotinic receptor of macrophages to reduce pro-inflammatory factors; colonic TNF-α mRNA expression is decreased, the inflammatory cascade is inhibited, intestinal epithelial cell apoptosis is reduced, tight junctions are protected, intestinal permeability is lowered, and the antigen leakage-induced inflammatory cycle is blocked. These mechanisms work together to alleviate chronic intestinal inflammation and relieve symptoms related to IBS (Alam and Chen, 2023). Regulating autonomic nerve function as a core dynamic mechanism is achieved through neuromodulation techniques that balance sympathetic and parasympathetic nerve activity. Techniques such as taVNS, TEA, and acupuncture can enhance vagal tone, inhibit excessive sympathetic activation, and improve gastrointestinal motility disorders: taVNS accelerates colonic transit and enhances antral motility, TEA restores gastric slow-wave rhythm and promotes gastric emptying, and acupuncture relieves intestinal spasms by regulating the vagal-sympathetic balance (Huang et al., 2022; Zhang et al., 2015; Liu et al., 2012). These regulations reduce abnormal gut motility (such as constipation or diarrhea), maintain normal motility patterns, and provide a dynamic basis for improving IBS symptoms related to bowel movement abnormalities.

## 3 Application of different neuromodulation techniques in IBS

### 3.1 Electroacupuncture (EA)

**Figure 1:**
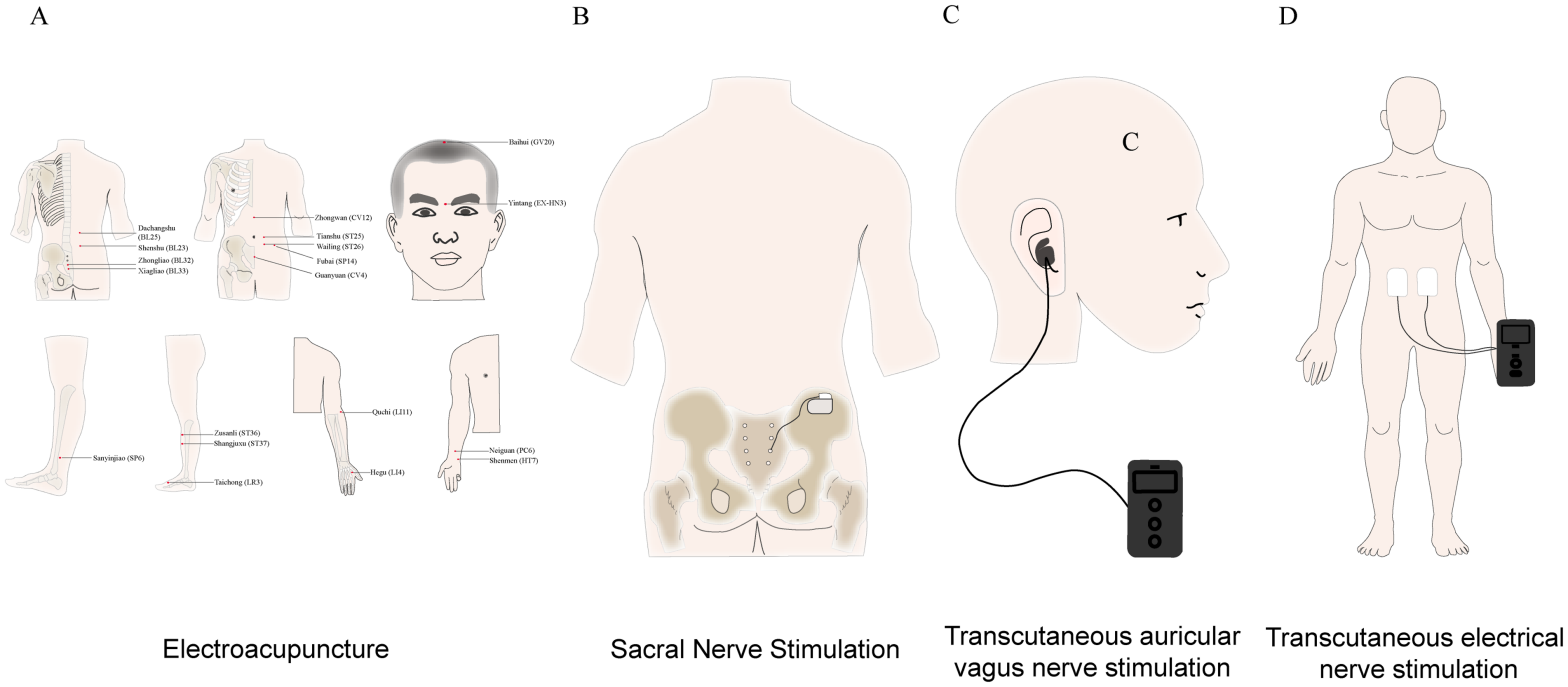
Neuromodulation technology schematic diagram. **(A)** Electroacupuncture (EA). **(B)** Sacral nerve stimulation (SNS). **(C)** Transcutaneous ear vagus nerve stimulation (taVNS). **(D)** Transcutaneous nerve electrical stimulation (TENS).

EA is a therapeutic modality that integrates traditional acupuncture with modern electrical stimulation. It applies a weak electrical current to acupuncture points based on traditional needling, thereby enhancing the stimulation of these points. The intensity of the electrical current is typically selected at a level that the patient can perceive and tolerate. The stimulation parameters, such as frequency, pulse width, and waveform, are of vital importance. For instance, low-frequency stimulation (e.g., 2 Hz) is often used to modulate the autonomic nervous system, high-frequency stimulation (e.g., 100 Hz) is utilized for pain relief, and frequencies in the range of 15–25 Hz are more effective for enhancing gastrointestinal motility. EA treatment usually lasts for 30 min, administered once daily or 3–4 times per week. It is recommended to perform EA after meals when gastrointestinal motility is more active (Table 2). Commonly used acupuncture points include Zusanli (ST36) and Neiguan (PC6), which are closely related to gastrointestinal function (Figure 1). EA devices can precisely control stimulation parameters, ensuring the consistency and reproducibility of treatment. During operation, it is necessary to ensure patient comfort and avoid excessive stimulation (Chen et al., 2018; Li et al., 2022). Multiple studies have demonstrated that EA is significantly effective in treating IBS and its related symptoms, particularly in alleviating abdominal pain, improving bowel motility frequency and stool consistency, and modulating brain-gut axis function. Peng et al. (2018) conducted an RCT involving 144 IBS patients, who were randomly assigned to four groups to receive EA at either the same or different acupoints. The results showed that EA was significantly effective in reducing abdominal pain intensity and performed well in improving IBS symptom severity scores. Zhao et al. (2015) compared the efficacy of EA and mild moxibustion in IBS-C patients and found that EA was more effective in improving bloating, bowel movement frequency, and stool consistency. The study also observed through fMRI technology that EA could significantly reduce abnormal activation in brain regions related to visceral pain and emotional processing in IBS patients during rectal distension stimulation, such as the anterior cingulate cortex (ACC), insula cortex (IC), and prefrontal cortex (PFC). Zhou et al. (2014) conducted a study involving 38 patients with constipation, including those with IBS-C, slow-transit constipation (STC), pelvic floor dyssynergia (PFD), and inadequate defecatory propulsion (IDP). The results showed that EA treatment could take effect within 5 days, demonstrating a rapid symptom relief feature. Zheng et al. (2016) conducted a multicenter randomized controlled trial, the results of which indicated that EA is comparable to loperamide in reducing bowel movement frequency in IBS-D patients and has significant effects on improving stool consistency and quality of life. However, the effectiveness of EA in treating IBS may be influenced by factors such as patient individual differences, treatment protocol selection, and placebo effects. Mak et al. (2019) failed to demonstrate the effectiveness of EA in patients with comorbid generalized anxiety disorder and IBS, which may be related to the clinical complexity of the patients or the selection of acupoints. The long-term effects and cost-effectiveness of EA treatment for IBS still need further research, and future studies need to further optimize EA treatment protocols and explore more effective acupoint combinations and stimulation parameters.

**Table 2:** Characteristics of the included studies on the therapeutic efficacy of neuromodulation in the treatment of irritable bowel syndrome.

First author (year)	Design	Type	Patients (n)	Technology	Comparison	Parameter	Protocol	Outcomemeasure	Results	Follow-up	Adverse event
Sun et al. (2021)	RCT	IBS-D	73 (36 vs. 37)	EA	EA	1 Hz, 4 ~ 6 mA	30 min, 6 times a week, for 4 weeks	IBS-SSS IBS-QOL HAMD	Overall efficiency: 94.4% (34/36) vs. 78.4% (29/37) (*p* < 0.05). IBS-SSS, HAMD, IBS-QOL (*p* < 0.05)	NA	NA
Peng et al. (2018)	RCT	IBS (abdominal pain)	144	EA	A, homotopic noxious B, homotopic innocuous C, heterotopic noxious D, heterotopic innocuous	2/100 Hz, noxious group has a pain threshold of 130%, non-noxious stimuli have a pain threshold of 70%	Homotopic acupoints Tianshu (ST25) and Wailing (ST26), Heterotopic acupoints Zusanli (ST36) and Shangjuxu (ST37) 30 min,14 sessions of treatment, twice per week for 7 weeks	VAS IBS-SSS IBS-QOL PT SCL-90	Trial protocol	3 m	NA
Zhao et al. (2015)	RCT	IBS-C	60 (30 vs. 30)	EA Mox	Self-control mutual control	EA: 2 Hz, 3.0 mA; Mox: acupoint surface temperature of 46 °C ± 1°C	Bilateral Tianshu (ST25) and Shangjuxu (ST37), 30 min, 6 times a week, for 4 weeks	VAS-IBS Bristol Stool Form Scale HAMA HAMD fMRI	GI symptoms: both groups improved in abdominal pain and bloating. EA outperformed MOX in relieving bloating, stool frequency, straining and consistency (*p* < 0.01). Psychological status: EA produced greater reductions in HAMA and HAMD scores than MOX (*p* < 0.01). Visceral sensitivity: EA raised pain threshold and lowered VAS at 100 and 150 mL rectal distension (*p* < 0.05). Brain activation: during 150 mL distension, EA significantly decreased voxel counts in ACC, right IC and PFC (*p* < 0.05–0.01), whereas MOX showed no change.	3 m	Mox group: 1 mild burn, no serious adverse events
Zhao et al. (2015)	RCT	IBS-D	60 (30 vs. 30)	EA Mox	Self-control mutual control	EA:2 Hz, 3.0 mA; Mox: acupoint surface temperature of 46 °C ± 1°C	Bilateral Tianshu (ST25) and Shangjuxu (ST37), 30 min, 6 times a week, for 4 weeks	VAS-IBS Bristol Stool Form Scale HAMA HAMD Immunohistochemistry fMRI	GI symptoms: both groups improved in pain and bloating. MOX surpassed EA in relieving urgency, frequency, and stool form (*p* < 0.01). Psychological state: MOX produced larger reductions in HAMA and HAMD than EA (*p* < 0.01). Neurotransmitters: both groups down-regulated colonic 5-HT, 5-HT3R, 5-HT4R (*p* < 0.01), with MOX showing a greater 5-HT decrease (*p* < 0.05). Brain activation: during 150 mL rectal distension, MOX significantly lowered voxel counts in left/right IC and PFC (*p* < 0.05–0.01), whereas EA only reduced PFC activity (*p* < 0.05).	3 m	Mox group: 1 mild burn, no serious adverse events
Zhou et al. (2014)	Prospective	IBS-C	38 (IBS-C = 9)	EA	Self-control mutual control	2 Hz/15 Hz	Tianshu (ST25), Fujie (SP14), Zusanli (ST36), Shangjuxu (ST37), Shenshu (BL23), Dachangshu (BL25), Zhongliao (BL33), Xialiao (BL34) 30 min, 5 times a week, for 2 weeks	Score of clinical symptoms of constipation	Onset time: IBS-C 1.78 ± 0.83 days, markedly shorter than STC 4.10 ± 1.85 days and PFD 4.30 ± 2.00 days (*p* < 0.05). IDP 3.11 ± 1.90 days, no difference vs. others. Symptom improvement: all subtypes showed significant total-score reductions at onset day (*p* < 0.05–0.01), with IBS-C greatest (2.33 ± 2.12) vs. other three (*p* < 0.05–0.01). Improvements focused on urge, frequency (STC > others, *p* < 0.01), incomplete evacuation/anal heaviness (IBS-C both, *p* < 0.05), and bloating/pain (IBS-C, STC, PFD. *p* < 0.05). No significant gains in straining, duration, or stool consistency (all *p* > 0.05).	NA	NA
Zheng et al. (2016)	Prospective	IBS-D FD	441	EA	He group Shu-Mu group He-Shu-Mu group Loperamide group	15 Hz, intensity to patient tolerance Loperamide: 2 mg t.i.d.	16 treatments in 4 weeks (10 in first 2 weeks, 6 in last 2 weeks). Loperamide group: 4-week course; stop after ≥3 consecutive days of normal stool.	Weekly bowel frequency Bristol Stool Scale Weekly normal-stool days SF-36	Bowel frequency: all groups decreased by 5.35 times per week after treatment (*p* > 0.05 between electro-acupuncture and loperamide). Stool form: all groups improved (*p* > 0.05). Normal-stool days: all groups increased (*p* > 0.05). Quality of life: all groups improved in every SF-36 domain (*p* > 0.05).	4w	11 adverse events: EA—syncope 3, insomnia 4, abdominal pain 1, cold limbs 1, fatigue 1; loperamide—hot flush 1; none severe.
Mak et al. (2019)	RCT	IBS-D GAD	80 (40 vs. 40)	EA	Sham	2 Hz, 0.5–1.5 mA	Bilateral Neiguan (PC6), Shenmen (HT7), Taichong (LR3), Sanyinjiao (SP6), Zusanli (ST36), Shangjuxu (ST37), plus Baihui (GV20) and Yintang (EX-HN3);30 min, once weekly for 10 sessions.	GAD7 Bristol stool scale PHQ-9 PHQ-15 The EuroQol-5 Dimensions	Anxiety: relief rate after 10 weeks and at 16-week follow-up did not differ between electro-acupuncture and sham groups (*p* = 0.06. *p* = 0.65). Bowel symptoms showed no difference (*p* > 0.05). Depression, somatic symptoms and quality of life also showed no difference (*p* > 0.05). Both groups improved, but inter-group differences were not significant (*p* > 0.05).	6w	NA
Fassov et al. (2014)	RCT	IBS-D IBS-M	20	SNM	Self-crossed control	14 Hz, 210 μs, 0.1–4.0 V	Open or close randomly based on the group, for each stage for one month	GSRS-IBS IBS-IS Bowel diaries	“ON” vs. “off”: GSRS-IBS (p = 0.0009). IBS-IS (*p* = 0.0003). Bowel habits: the frequency of defecation, the number of episodes of urgency, and the time spent on toilet use per week have all significantly decreased (*p* = 0.0031,0.0180,0.0184) 1 year follow-up: GSRS-IBS and IBS-IS (*p* = 0.0001)	12 m	7 patients reported 10 equipment-related adverse events (4 were mild; 1 was moderate; and 5 were severe)
Fassov et al. (2025)	Prospective	IBS-D IBS-M	36	SNM	Self-control	Based on the individual response of the patient	Permanent implantation	GSRS-IBS IBS-IS	Symptom improvement: - 5-year follow-up (*p* < 0.0001), - 10-year follow-up (*p* = 0.0007), Quality of life: - 5-year follow-up (*p* < 0.0001), - 10-year follow-up (*p* = 0.0002), Treatment success rate: 10-year follow-up,77% of patients achieved treatment success.	1-10y	56 adverse events, 5 cases where devices were removed due to adverse events
Fassov et al. (2019)	RCT	IBS-D IBS-M	21	SNM	Self-crossed control	Sub-sensory: 90% of the optimal perception intensity. Supra-sensory: parameters set based on the patient’s optimal perception response.	Participants were randomly assigned to receive sub-sensory stimulation or to be deprived of it, for 2 weeks each stage	GSRS-IBS IBS-IS Bowel diaries	Sub-sensory stimulation vs. Off: - Total GSRS-IBS score (*p* = 0.0572) - Pain score (*p* = 0.0188) - Daily bowel movement frequency (*p* = 0.0373) Super-sensory stimulation vs. Off: - Total GSRS-IBS score (*p* = 0.0017) - IBS-IS score (*p* = 0.0325) - Frequency of defecation and episodes of urgency (*p* = 0.0149, *p* = 0.0114) Placebo effect:52% of patients experienced a placebo response, with an overall median placebo effect of 14%	NA	NA
Shi et al. (2021)	RCT	IBS-C	42 (21 vs. 21)	taVNS	Sham-taVNS	25 Hz, 0.5 ms, 2 s on/3 s off, 0–2 mA.	taVNS: bilateral cymba concha, 30 min twice daily at 08:00 and 20:00 for 4 weeks. Sham: same schedule, electrodes on non-acupoint elbow area.	CSBMs/week VAS IBS-SSS IBS-QOL BSFS SAS HRAM Autonomic function Lab markers	Constipation: taVNS improved CSBMs/week (*p* < 0.001) and BSFS vs. sham (*p* = 0.001). Pain: VAS improved (*p* < 0.001) vs. sham (*p* = 0.001). Overall: IBS-SSS lower, IBS-QOL higher, anxiety and depression lower (all *p* < 0.001). Rectal: RAIR threshold lower (*p* = 0.001), sensory threshold lower (*p* < 0.04). Mechanism: vagal tone higher (*p* = 0.04); TNF-α, IL-6, 5-HT lower (*p* < 0.05). Vagal tone correlated with CSBMs (*r* = 0.391) and inversely with VAS (*r* = −0.347).	NA	No serious adverse events; sham group 1 mild skin allergy from electrode, self-resolved.
Krasaelap et al. (2020)	RCT	IBS-C IBS-D IBS-M	50 (27 vs. 23)	PENFS	Sham	3.2 V rectangular pulses, 1 Hz/10 Hz alternating, 2 h on / 2 h off.	5 days weekly for 4 weeks.	PFSD SRS STAI-C FDI	Pain relief: PENFS superior to sham (*p* = 0.024). Composite pain score change: PENFS 7.5, sham 14.4 (*p* = 0.026). Overall symptoms: PENFS superior (*p* ≤ 0.001). At 8–12-week follow-up: no difference (*p* = 0.33).	8-12w	One PENFS patient had adhesive allergy, completed treatment; no serious events.
Nojkov et al. (2024)	RCT	IBS-C	19	TEA	Sham-TEA	TEA ST36 or PC6: 100 Hz, 0.5 ms, on 0.1 s/off 0.4 s; TEA ST36 or PC6: 25 Hz, 0.5 ms, on 2 s/off 3 s; Rectal distension: 5–50 mmHg, 30 s each, 30 s deflation.	Five 15-min randomized sessions on separate days: TEA/ST36-100 Hz, bilateral ST36; TEA/ST36-25 Hz, bilateral ST36; TEA/PC6-100 Hz, bilateral PC6; TEA/PC6-25 Hz, bilateral PC6; Sham, non-acupoint, 25 Hz.	VAS Rectal sensory threshold HRV Rectal compliance	Pain relief: TEA/ST36-100 Hz lowered VAS (*p* < 0.04) versus sham (*p* = 0.04). Rectal sensation: TEA/ST36-100 Hz raised first sensation (*p* = 0.007) and urge threshold (*p* = 0.026). Autonomic function: TEA/ST36-100 Hz decreased sympathetic (*p* < 0.03) and increased parasympathetic activity (*p* < 0.04). Other interventions: only TEA/PC6-25 Hz improved first sensation. Remainder nonsignificant (*p* > 0.05).	NA	Mild adverse events: transient rectal bleeding 2, post-stim abdominal pain worsened 3 (1 withdrew), belching 1; none serious.
Hu et al. (2022)	RCT	IBS-D	42 (21 vs. 21)	TEA	Sham-TEA	LI4: 100 Hz, 0.1 s/0.4 s train, 0.5 ms; ST36: 25 Hz, 2 s/3 s train, 0.5 ms.	TEA: Hegu (LI4, right hand) & Zusanli (ST36, left leg), 30 min twice daily after meals for 1 month. Sham: same schedule, no current.	VAS IBS-SSS IBS-QOL Lab markers	Pain relief: TEA reduced VAS more than sham (*p* = 0.014). IBS-QOL improved in TEA (*p* < 0.0001), unchanged in sham. IBS-SSS decreased in both groups, ns (*p* > 0.05). Dropout higher in sham (*p* = 0.021). NE, PP, IL-6, IL-10 unchanged (*p* > 0.05).	NA	TEA: no adverse events. Sham: 1 mild skin allergy from electrode, no impact.
Çoban et al. (2012)	RCT	IBS-C IBS-D IBS-M	58 (29 vs. 29)	IFC	Sham	Carrier 4 kHz, beat 80–150 Hz, 15–25 mA.	IFC: abdomen and T9-L2 paraspinal, four crossed electrodes, 15 min three times weekly for 4 weeks. Sham: same placement, vacuum only, no current.	IBS-GAI numeric rating scale VAS IBS-QOL	Symptoms: IFC reduced pain, bloating, bowel sounds at end and 1 month later (*p* < 0.05), with further improvement at 1 month (*p* < 0.01); sham showed no added benefit (*p* > 0.05). IBS-QOL: IFC improved total score (*p* = 0.014) and anxiety, health worry, food avoidance domains (*p* < 0.05). Response: IFC had higher pain relief rate at 1 month than sham (*p* < 0.05).	1 m	NA
Wang and Liu (2018)	Retrospective	IBS-D	108 (54 vs. 54)	NMES	Underwent DHC alone	2–100 Hz	Combined: DHC 80 mg orally twice daily for 4 weeks plus NMES (bilateral Shangjuxu (ST37) and Tianshu (ST25), 20 min twice weekly for 4 weeks). Control: DHC only.	VAS Weekly bowel movements Bristol scale	Pain: no difference (*p* = 0.14). Bowel frequency: no difference (*p* = 0.42). Bristol score: similar improvement (*p* = 0.71).	NR	Mild nausea, headache, dizziness, fatigue in both groups; no difference (P > 0.05); no deaths.
Lind et al. (2015)	RCT	IBS-D IBS-M	10	SCS	Self-crossed control	50 Hz, 200–500 μs pulse width, 1.3–3.3 V amplitude.	Under local anesthesia, Quad-plus lead (Medtronic) placed epidurally at T5–T8, connected to abdominal Itrel-3 generator; stimulation 8–12 h daily, total 28 w (2 w run-in, 6 w crossover, 12 w continuous, 2 w washout).	VAS Daily diary HADS GSRS-IBS QoL	Pain: VAS 7 to 3–4 during stimulation (*p* < 0.03–0.04), attacks fewer. IBS-D: diarrhea reduced in some, not significant (*p* > 0.05). Long-term: 6 kept device. After 61 months 3 still use SCS with stable relief.	18-78 m	Mild adverse events: fatigue 2, gait instability with leg numbness 2, excessive leg stimulation and implant-site pain 1; no serious complications.
Algladi et al. (2015)	RCT	IBS-C IBS-D	26 (10 vs. 16)	TMS LSMS	Sham	LSMS: 1 Hz, TMS: 10 Hz, 600 pulses	Two active stimulations (1 Hz rLSMS, 10 Hz rTMS) and one sham.	HAD IBS-SSS VAS Brief McGill Pain Questionnaire	Healthy: 1 Hz rLSMS raised rectal pain threshold and anal sensory threshold for 1 h. 10 Hz rTMS raised rectal pain threshold and improved anal pain threshold for 1 h. IBS: both 1 Hz rLSMS and 10 Hz rTMS increased rectal pain threshold for 1 h. 10 Hz rTMS also improved anal pain. 1 Hz rLSMS raised rectal sensory threshold. Sham had no effect.	NA	NA

CSBMs/week, Complete spontaneous bowel movements per week; EA, Electroacupuncture; FD, Functional diarrhea; FDI, Functional Disability Inventory; GAD, Generalized anxiety disorder; GSRS-IBS, Gastrointestinal Symptom Rating Scale-Irritable Bowel Syndrome; HAMA, Hamilton Anxiety Scale; HAD, Hospital Anxiety and Depression; HAMD, Hamilton Depression Rating Scale; HRAM, High-resolution anorectal manometr; HRV, Heart rate variability; IBS, Irritable bowel syndrome; IBS-IS, Irritable Bowel Syndrome Impact Scale; IBS-QOL, Irritable Bowel Syndrome Quality of Life Questionnaire; IBS-SSS, Irritable Bowel Syndrome Symptom Severity Scale; IFC, Interferential current therapy; LSMS, Lumbosacral Magnetic Stimulation; Mox, Moxibustion; NMES, Neuromuscular Electrical Stimulation; NR, Not recorded; PENFS, Percutaneous electrical nerve field stimulation; PFSD, Pain Frequency-Severity-Duration Scale; PHQ, Patient Health Questionnaire; PT, Pain threshold; RCT, Randomized controlled trial; SCL-90, Symptom Checklist-90; SCS, Spinal cord stimulation; SF-36,36-item short-form health survey; SNM, Sacral Neuromodulation; SRS, Symptom Response Scale; STAI-C, State–Trait Anxiety Inventory for Children; TAVNS, Transcutaneous Auricular Vagal Nerve Stimulation; TEA, Transcutaneous electrical acustimulation; TMS, Transcranial Magnetic Stimulation; VAS, Visual analog scale.

### 3.2 Sacral nerve stimulation (SNS)

SNS involves a minimally invasive surgical procedure to implant electrodes into the sacral foramen (S3 or S4) to continuously stimulate the sacral nerves. The stimulation parameters (frequency, pulse width, intensity) are adjusted based on patient response and are typically set slightly above the sensory threshold to ensure efficacy. Short-term stimulation is used for rapid assessment of efficacy, while long-term stimulation is achieved through the implantation of a permanent neurostimulator (Fassov et al., 2017). A 2-month study enrolled 21 patients with IBS-D or IBS-M. The results showed that SNS significantly reduced the IBS-specific symptom scores (*p* = 0.0009) and significantly improved the quality of life scores (*p* = 0.0003). Follow-up at 1 year found that both the IBS symptom scores and quality of life scores were significantly better than baseline levels (*p* < 0.0001) (Fassov et al., 2014). Another 6-week study enrolled 21 IBS patients. The results showed that during subsensory stimulation, the IBS symptom scores were reduced (*p* = 0.0572), pain was significantly alleviated (*p* = 0.0188), and the number of daily bowel movements was significantly decreased (*p* = 0.0373). During suprasensory stimulation, both the IBS symptom scores and quality of life scores were significantly improved (*p* < 0.05) (Fassov et al., 2019). In terms of long-term efficacy, Fassov et al. (2025) conducted a prospective cohort study following 36 IBS patients who underwent SNS treatment for up to 10 years. The results showed that at the 5-year and 10-year follow-ups, both the IBS symptom scores (GSRS-IBS) and quality of life scores (IBS-IS) were significantly improved (*p* < 0.0001 and *p* = 0.0002). Despite 56 adverse events occurring, most were corrected through reprogramming or minor surgeries.

### 3.3 Transcutaneous auricular vagus nerve stimulation (taVNS)

taVNS primarily regulates the autonomic nervous system and improves gastrointestinal function by stimulating the auricular branches of the vagus nerve. The stimulation is typically delivered using a small, portable device placed on specific areas of the ear, such as the helix or tragus, with a frequency generally ranging from 0.5 to 30 Hz. Each treatment session lasts about 10–30 min and is conducted 1–2 times per day (Song et al., 2023). A prospective, randomized, double-blind, controlled trial targeting adolescent IBS patients enrolled 51 patients, who were randomly assigned to receive either taVNS or sham stimulation. The results showed that 59% of patients in the taVNS group experienced a 30% or greater reduction in the intensity of their most severe abdominal pain after 3 weeks, compared to only 26% in the sham stimulation group (*p* = 0.024). Moreover, both the abdominal pain composite score and the IBS quality of life score were significantly improved in the taVNS group (Krasaelap et al., 2020). Another single-center, single-blind, randomized controlled study targeting adults enrolled 42 patients with IBS-C. The taVNS group received treatment for 4 weeks. The results indicated that taVNS significantly increased complete spontaneous bowel movements (CSBMs), reduced visual analog scale (VAS) pain scores, and improved IBS quality of life scores. It also significantly decreased the levels of pro-inflammatory cytokines TNF-α and IL-6 in serum, as well as 5-hydroxytryptamine (5-HT) levels in plasma, while enhancing vagal activity (Shi et al., 2021). These two studies demonstrate that taVNS can significantly improve abdominal pain and quality of life in both adolescent and adult IBS patients, and also significantly increase bowel movement frequency in IBS-C patients. The mechanisms may involve the vagus nerve’s regulation of gut function and its suppression of inflammatory responses.

### 3.4 Transcutaneous electrical nerve stimulation (TENS)

TENS is a non-invasive technique that delivers electrical current through surface electrodes on the skin to stimulate nerve fibers, thereby alleviating pain and improving gastrointestinal motility. Each treatment session lasts about 30–60 min and is conducted 1–2 times per day, and it can be self-administered at home (Nojkov et al., 2024). TENS shows certain application prospects in the field of IBS treatment, although its efficacy varies in different situations. Hu et al. (2022) provided TENS treatment for 42 patients with IBS-D for 1 month. The results showed that TENS significantly reduced the patients’ abdominal pain scores (*p* = 0.014) and significantly improved quality of life (*p* < 0.0001), while the control group receiving sham stimulation did not show significant changes. This indicates that TENS has potential positive effects in alleviating abdominal pain and improving quality of life in IBS-D patients. Çoban et al. (2012) provided TENS treatment for 42 patients with IBS-C for 4 weeks. The results showed that TENS significantly increased CSBMs (*p* < 0.001), reduced VAS pain scores (*p* = 0.001), and improved quality of life (*p* = 0.020), while the sham stimulation group did not show significant improvement. These results further confirm the effectiveness of TENS in alleviating symptoms and improving quality of life in IBS-C patients. However, in some cases, the effect of TENS as an adjunctive therapy may not be significant. Wang and Liu (2018) provided TENS treatment for 108 IBS-D patients for 4 weeks, with 54 patients receiving TENS as an adjunct to the antispasmodic dicyclomine hydrochloride, and the other 54 patients receiving only dicyclomine hydrochloride. The results showed that TENS as an adjunctive therapy did not significantly improve abdominal pain (*p* = 0.14) or average weekly bowel movements (*p* = 0.42) in IBS-D patients, with no significant difference compared to dicyclomine hydrochloride alone. Moreover, the impact of TENS on autonomic nerve function and inflammatory cytokines is limited. Additionally, some studies have small sample sizes and short treatment durations, which may affect the generalizability of the results.

### 3.5 Others

In addition to EA, SNS, taVNS, and TENS, several other neuromodulation techniques have been employed in the treatment of IBS. For instance, non-invasive magnetoelectric neurostimulation techniques such as transcranial magnetic stimulation (TMS) and lumbar-sacral magnetic stimulation (LSMS) have been shown to modulate visceral sensitivity in both healthy individuals and IBS patients (Algladi et al., 2015). Furthermore, SCS has also been utilized for IBS treatment. By implanting electrodes at the T5-T8 level, SCS can significantly reduce abdominal pain scores in IBS patients (Lind et al., 2015). These studies suggest that neuromodulation techniques may alleviate IBS symptoms by influencing the brain-gut axis and visceral sensitivity, offering new ideas and approaches for the treatment of IBS.

## 4 Discussion

In recent years, the application of neuromodulation techniques in the treatment of IBS has been increasing, especially in improving gastrointestinal motility disorders and abdominal pain (Engeler et al., 2013). taVNS, by stimulating the vagus nerve, can modulate the brain-gut axis and improve gut function. It has shown promising effects in some clinical trials, particularly in alleviating symptoms such as abdominal pain and diarrhea. SNS, a method of regulating gut function by stimulating the sacral nerves, has demonstrated significant efficacy in patients with IBS-C. This technique is believed to improve gut motility and relieve constipation symptoms (Miller et al., 2019). EA, as a non-invasive treatment method, has also been gradually applied to IBS treatment due to its ability to regulate nerve and muscle functions. EA can improve gut motility and alleviate abdominal pain by precisely stimulating acupoints related to the gut. Compared with traditional pharmacological treatments, EA has a longer-lasting therapeutic effect and can be self-administered at home, which increases the convenience of treatment and patient compliance (Southwell, 2020).

Neuromodulation techniques have emerged as a novel treatment for IBS, especially for patients who do not respond well to traditional pharmacological therapies (Xiang et al., 2022). By directly modulating the nervous system to improve gastrointestinal motility and alleviate abdominal pain, these techniques hold significant clinical potential. One of their main advantages is the ability to target the pathophysiological mechanisms of IBS, particularly gastrointestinal motility disorders and visceral pain relief. These techniques work by stimulating the central or peripheral nervous system to improve gut function and modulate the brain-gut axis, thereby achieving therapeutic effects (Yin and Chen, 2023). Additionally, as non-pharmacological treatments, neuromodulation techniques can avoid the side effects of medications, providing an effective alternative for patients who do not respond well to or cannot tolerate drugs. In terms of cost, Shah et al. (2024) demonstrated that TENS treatment for pediatric IBS is more cost-effective compared to traditional treatments. TENS can save $4,744 for insurance companies and $5,802 for patient families within one year, while also increasing 18 healthy days and 0.05 Quality-Adjusted Life Year.

However, neuromodulation techniques also have certain limitations and disadvantages. First, although most neuromodulation treatments are relatively safe, there are still some side effects and risks. For example, invasive treatments such as SNS require surgical implantation of electrodes, which may bring problems such as infection, device failure, or lack of durability of the effect (Gefen et al., 2024). In addition, non-invasive treatments such as EA or taVNS may have unstable effects when there are large individual differences, and the treatment plan needs to be adjusted according to the needs of different patients. Second, the therapeutic effect of neuromodulation techniques may be closely related to the stimulation parameters, so it is necessary to finely adjust the treatment frequency and intensity, which increases the complexity of treatment and the demand for individualization (Chang et al., 2024).

Regarding the diagnostic criteria for IBS, the current standards have shifted from the Rome III to the Rome IV criteria. The primary differences between the Rome III and Rome IV criteria for diagnosing IBS lie in the definition and the frequency requirements for symptoms. The Rome IV criteria have eliminated the vague term “abdominal discomfort,” focusing solely on “abdominal pain” as the core symptom. Additionally, the frequency requirement for abdominal pain has been increased from at least 3 days per month in Rome III to at least 1 day per week. These changes make the diagnosis more stringent and precise, reducing the likelihood of misdiagnosis and missed diagnoses (Black et al., 2020b). Studies have shown that the specificity of the Rome IV criteria reaches 82.9%, significantly higher than the 65.0% of Rome III, with a higher positive likelihood ratio, indicating a substantial improvement in diagnostic accuracy (Vork et al., 2018). For patients, the Rome IV criteria not only enhance diagnostic accuracy but also place greater emphasis on the psychological state of patients. This helps physicians to pay attention to patients’ mental health while treating IBS, providing more comprehensive therapeutic support. For scientific research, the Rome IV criteria enhance the homogeneity of study populations, increasing the reliability and reproducibility of research findings and promoting in-depth studies of the pathophysiological mechanisms of IBS and the optimization of treatment methods (Black et al., 2021).

Neuromodulation for the treatment of IBS is still in the experimental stage, but its developmental trajectory from initial exploration to controlled trials and then to elucidation of mechanisms has already demonstrated potential value for refractory IBS. With a deeper understanding of the pathogenesis of IBS, the combination of neuromodulation and gut microbiota therapy shows great potential. Ford et al. (2024) proposed that chronic visceral pain is a disease of the microbiota-gut-brain axis, with both peripheral and central mechanisms involved in its pathogenesis. Future research can further explore the role of neuromodulation in this axis, focusing on the development of personalized treatment plans. This includes in-depth investigation of how neuromodulation affects the gut microbiota and its metabolites, thereby changing IBS symptoms. Identifying biomarkers that predict treatment response and stratifying patients based on factors such as gender, age, and IBS subtype may achieve precise treatment. Multimodal treatment strategies, such as combining neuromodulation, microbiota modulation, and psychological interventions, as well as developing drugs that act on both the nervous system and the microbiota, will provide patients with more comprehensive treatment options (Zhao et al., 2025). In terms of technological innovation, the application of non-invasive neuromodulation techniques and smart devices will further improve the acceptability and adherence to treatment. Large-scale multicenter studies are also needed for further validation. At present, there are still few studies on the long-term efficacy and mechanisms of neuromodulation in the treatment of IBS. Although initial clinical trial results support its effectiveness, there is a lack of sufficient large-scale, long-term clinical data to prove its wide applicability.

## 5 Conclusion

Neuromodulation techniques provide an innovative approach for the treatment of IBS. As a gut-brain interaction disorder, IBS has limitations in traditional treatments, such as insufficient targeting of core mechanisms and significant side effects. Neuromodulation, through electrical and electromagnetic means, can regulate neural pathways to specifically improve abnormalities in the brain-gut axis and visceral hypersensitivity, which are key pathological aspects, effectively alleviating symptoms such as abdominal pain and abnormal bowel movements. Current techniques such as EA, SNS, taVNS and TENS have shown positive effects in clinical trials, improving patients’ quality of life, with some techniques also having cost-effectiveness advantages. There are still limitations in this field; invasive techniques carry certain risks, while the efficacy of non-invasive techniques is influenced by individual differences and parameters, and long-term data need to be supplemented. In the future, there is a need to focus on combined treatments, personalized plans, technological innovation, and large-scale studies to fully realize its potential value for refractory IBS and promote it as an important part of comprehensive IBS management.

## Glossary

IBS: Irritable Bowel Syndrome
CNS: Central Nervous System
HPA: Hypothalamic–Pituitary–Adrenal
SCFAs: Short-Chain Fatty Acids
TJ: Tight Junction
OCLN: Occludin
ZO: Zonula Occludens
CLDN: Claudin
MLCK: Myosin Light Chain Kinase
MLC: Myosin Light Chain
HAPC: High-Amplitude Propulsive Contractions
IBS-D: Irritable Bowel Syndrome with Diarrhea
IBS-C: Irritable Bowel Syndrome with Constipation
IBS-M: Irritable Bowel Syndrome with Mixed Bowel Habits
IBS-U: Unspecified Irritable Bowel Syndrome
EA: Electroacupuncture
SNS: Sacral Nerve Stimulation
taVNS: Transcutaneous Auricular Vagus Nerve Stimulation
TENS: Transcutaneous Electrical Nerve Stimulation
VNS: Vagus Nerve Stimulation
RCT: Randomized Controlled Trial
fMRI: Functional Magnetic Resonance Imaging
ACC: Anterior Cingulate Cortex
IC: Insula Cortex
PFC: Prefrontal Cortex
STC: Slow-Transit Constipation
PFD: Pelvic Floor Dyssynergia
IDP: Inadequate Defecatory Propulsion
CSBMs: Complete Spontaneous Bowel Movements
VAS: Visual Analog Scale
TMS: Transcranial Magnetic Stimulation
LSMS: Lumbar-Sacral Magnetic Stimulation
SCS: Spinal Cord Stimulation
GSRS-IBS: Gastrointestinal Symptom Rating Scale-Irritable Bowel Syndrome
IBS-IS: Irritable Bowel Syndrome Impact Scale
TEA: Transcutaneous Electrical Acustimulation
FODMAP: Fermentable Oligosaccharides, Disaccharides, Monosaccharides and Polyols
